# Characterization of a Highly Thermostable and Organic Solvent-Tolerant Copper-Containing Polyphenol Oxidase with Dye-Decolorizing Ability from *Kurthia huakuii* LAM0618^T^

**DOI:** 10.1371/journal.pone.0164810

**Published:** 2016-10-14

**Authors:** Xiang Guo, Shan Zhou, Yanwei Wang, Jinlong Song, Huimin Wang, Delong Kong, Jie Zhu, Weiwei Dong, Mingxiong He, Guoquan Hu, Zhiyong Ruan

**Affiliations:** 1 Institute of Agricultural Resources and Regional Planning, Chinese Academy of Agricultural Sciences, Beijing, China; 2 Biogas Institute of Ministry of Agriculture, Chengdu, China; 3 Chinese Academy of Fishery Sciences, Beijing, China; Istituto di Genetica Molecolare, ITALY

## Abstract

Laccases are green biocatalysts that possess attractive advantages for the treatment of resistant environmental pollutants and dye effluents. A putative laccase-like gene, *laclK*, encoding a protein of 29.3 kDa and belonging to the Cu-oxidase_4 superfamily, was cloned and overexpressed in *Escherichia coli*. The purified recombinant protein LaclK (LaclK) was able to oxidize typical laccase substrates such as 2,6-dimethoxyphenol and l-dopamine. The characteristic adsorption maximums of typical laccases at 330 nm and 610 nm were not detected for LaclK. Cu^2+^ was essential for substrate oxidation, but the ratio of copper atoms/molecule of LaclK was determined to only be 1:1. Notably, the optimal temperature of LaclK was 85°C with 2,6-dimethoxyphenol as substrates, and the half-life approximately 3 days at 80°C. Furthermore, 10% (v/v) organic solvents (methanol, ethanol, isopropyl alcohol, butyl alcohol, Triton x-100 or dimethyl sulfoxide) could promote enzymatic activity. LaclK exhibited wide-spectrum decolorization ability towards triphenylmethane dyes, azo dyes and aromatic dyes, decolorizing 92% and 94% of Victoria Blue B (25 μM) and Ethyl Violet (25 μM), respectively, at a concentration of 60 U/L after 1 h of incubation at 60°C. Overall, we characterized a novel thermostable and organic solvent-tolerant copper-containing polyphenol oxidase possessing dye-decolorizing ability. These unusual properties make LaclK an alternative for industrial applications, particularly processes that require high-temperature conditions.

## Introduction

Laccases are copper-containing polyphenol oxidoreductase enzymes that belong to the multicopper oxidase family [1]. Laccases can catalyze the oxidation of a considerable number of phenolic and non-phenolic compounds while reducing molecular oxygen to water [2]. Four copper atoms per monomer are believed to be essential for laccase catalytic activity. The amino acid sequences of the copper atom binding domains are generally conserved and specifically contain the following four histidine-rich copper binding motifs: HXH, HXHG, HXXHXH and HCHXXXHXXXXM/L/F [3]. Based on their spectroscopic properties, the four copper atoms can be assigned to three types of copper centers: type 1, which is responsible for the blue color of laccases with an absorption maximum of approximately 610 nm; type 2, which is nearly undetectable; and type 3, which has an absorption maximum of approximately 330 nm [2]. However, not all laccases demonstrate a characteristic adsorption maximum of approximately 330 nm or 610 nm, and not all possess four copper atoms. For instance, the laccase from the γ-proteobacterium JB exhibits no maximum at 330 nm [4]. The “white” laccase POXA1 from *Pleurotus ostreatus* lacks of a typical absorbance maximum at 610 nm, and the metal content of this laccase comprises a single copper atom, two zinc atoms, and one iron atom [5]. The “yellow” laccase from *Sclerotinia sclerotiorum* contains four copper atoms, but a 610-nm maximum is not detectable [6]. The molecular weight of a regular three-domain laccase is usually 50–70 kDa or larger, whereas certain two-domain laccases, which lack the second domain, generally have a smaller molecular size (30–40 kDa) [7]. Interestingly, RL5, a four-copper laccase (28 kDa per monomer) from the bovine rumen, lacks the four characteristic histidine-rich copper binding motifs but exhibits much higher activity than typical laccases [8].

Laccases are regarded as a type of green and environmentally friendly biological catalyst and have received a great deal of attention in the context of industrial applications, such as pulp delignification and bleaching, textile wastewater decolorization, and food improvement [9]. Industrial processes usually include harsh conditions such as high temperature, acidic or alkaline pH, high salt and detergents; thus, laccases that are resistant to these conditions are preferable [10, 11]. An increasing number of studies have demonstrated that bacterial laccases possess greater advantages than laccases of fungal origin. For example, the *Tth* laccase from *Thermus thermophilus* exhibits extreme stability against heat with a half-life of more than 14 h at 80°C, making it the most thermophilic laccase reported so far [12]. The Ssl1 laccase from *Streptomyces sviceus* is highly alkali-stable and resistant to detergents and organic solvents [11]. Additionally, the SN4 laccase from *Bacillus tequilensis* is thermo-alkali-stable and metal-tolerant [13]. Bioinformatics analysis has demonstrated the high diversity of laccase or laccase-like enzymes in bacteria [14], but bacterial laccase-like enzymes have yet to be exploited as promising laccase resources.

*Kurthia* species have demonstrated their potential use in many applications, particularly in the decolorization of triphenylmethane dyes, textiles and dye-stuff effluents [15]. In our previous studies, *K*. *huakuii* LAM0618^T^ was isolated from biogas slurry [16], and further investigation identified its capacity to decolorize malachite green and degrade cinosulfuron [17]. Available genomic data [18] showed that *K*. *huakuii* LAM0618^T^ contains a putative protein sequence annotated as a “multi-copper polyphenol oxidoreductase laccase”. Bioinformatic analysis suggested that this gene may represent a novel bacterial laccase-like protein with heat-resistant properties.

In this study, a putative laccase-like gene (designated *laclK*) from *K*. *huakuii* LAM0618^T^ was cloned and heterologously expressed in *E*. *coli* based on a genome mining approach. The physicochemical properties of the recombinant LaclK protein (LaclK) and its ability to decolorize different dyes were investigated.

## Results

### Sequence analysis of *laclK*

**Fig 1:**
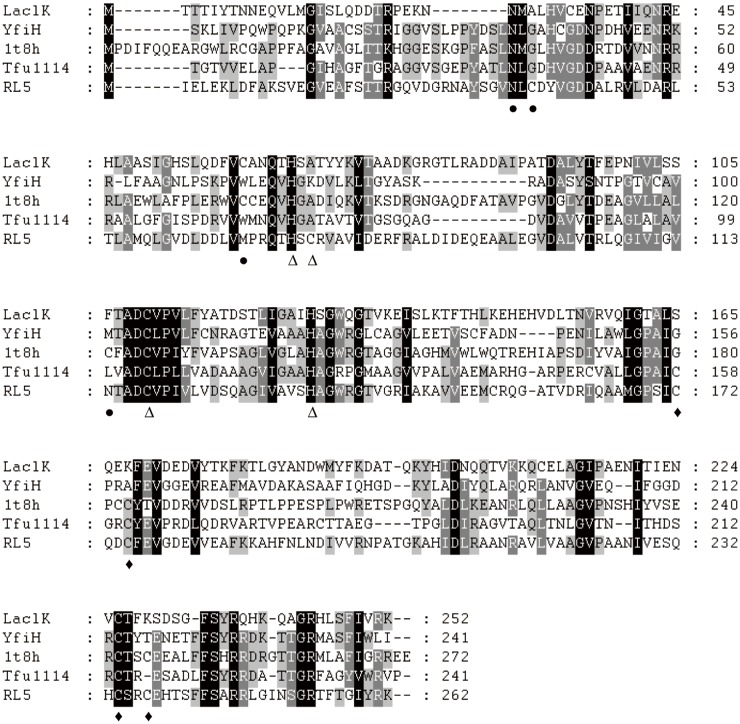
Multiple sequence alignment of LaclK with selected LaclK-related proteins. Amino acid sequences were retrieved from NCBI or the UniProt database. LaclK, a copper oxidase in this study; YfiH, a laccase from *E*. *coli* (UniProt accession no. P33644); 1t8h, an uncharacterized protein from *Bacillus stearothermophilus* (UniProt accession no. P84138); Tfu1114, a copper oxidase from *Thermobifida fusca* NTU10-1 (NCBI accession no. WP_011291561); RL5, a laccase from the bovine rumen metagenome (NCBI accession no. AM269758). The amino acid residues binding to the three copper sites in RL5 are indicated with ●, Δ, and ♦.

The putative ORF of *laclK* encodes a protein of 252 amino acids with a predicted molecular mass of 29,260 Da. Putative conserved domains were detected, demonstrating that LaclK belongs to the Cu-oxidase_4 superfamily. It should be noted that the Cu-oxidase_4 superfamily has been assigned to the multi-copper polyphenol oxidoreductase laccase protein family according to the Pfam database (Pfam PF02578). Similar sequence searching in BlastP indicated that LaclK is most similar to hypothetical proteins deduced from the genomes of *Kurthia massiliensis* (WP_010290511) and *Kurthia sp*. JC8E (WP_010308194), with 80% and 73% identity, respectively. Further analysis demonstrated that LaclK demonstrates high homology to two uncharacterized laccases from *Bacillus* sp. FJAT-22090 (WP_053591188) and *Planomicrobium glaciei* (WP_036811027) and a hypothetical protein from *Ureibacillus thermosphaericus* (WP_016838508), with 64%, 63% and 63% identity, respectively. Multiple amino acid sequence alignment with LaclK-related proteins indicated that LaclK shares 26.8%, 27.2%, 25.4% and 26.8% identity with YfiH, 1t8h, Tfu1114 and RL5. Among the 12 identified copper sites in RL5 [8], only five (N^36^, H^73^, C^118^, H^135^ and C^234^) were found to be conserved in LaclK (Fig 1). Both YfiH and 1t8h belong to the Cu-oxidase_4 superfamily. YfiH was identified as laccase [8], while 1t8h is an uncharacterized protein whose crystal structure is available (Pfam domain PF02578). Tfu1114 and RL5, like LaclK, have small molecular masses compared to other reported laccases and were previously identified as a copper-containing oxidase and a laccase, respectively [8, 19].

The aliphatic index is considered positively correlated with protein thermostability, and the indices of thermostable proteins are much higher than those of ordinary proteins [20]. The instability index is predictive of protein stability characteristics. If the value is below 40, the protein is likely stable in vitro assays. If the value is above 40, the protein is likely not stable [21]. Aliphatic index and instability index analyses for LaclK and certain previously reported heat-resistant laccases or laccase-like oxidases are summarized in Table 1. Recombinant LaclK exhibited a relatively high aliphatic index (75.62) and a low instability index (31.92).

**Table 1:** Aliphatic index and instability index analysis of LaclK compared with reported heat-resistant laccases or laccase-like oxidases.

Type	Aliphatic index	Instability index	Properties	Molecular weight (kDa)	NCBI accession no.
LaclK	75.62	31.92	This study	30	WP_029500662
Tfu1114	86.96	30.72	half-life at 90°C was 4.7 h; stable against organic solvents [19]	24.7	WP_011291561
EpoA	66.19	34.70	retained 40% activity after incubation at 70°C for 60 min [41]	114 (homotrimer)	BAB64332
SLAC	61.81	29.63	highly stable under alkaline, boiling and SDS treatment [42]	69 (dimer)	CAB45586
Ssl1	67.82	31.78	half-life at 60°C was 88 min; stable against alkaline, detergents and organic solvents [11]	32.5	EDY55866
CotA	77.89	45.51	half-life at 80°C was 112 min [35]	65	P07788
REN-7	79.15	42.86	half-life at 70°C was 100 min [40]	73	BAC16804
*Tth*	96.39	42.21	half-life at 80°C was 868 min [12]	53	AAS81712
TtMCO	81.61	47.45	half-life at 70°C was 2.24 days; half-life at 80°C was 350 min [30]	60	UniProt accession no. D1CEU4

Other laccases or laccase-like oxidases originating from different organisms used for comparison were *Thermobifida fusca* Tfu1114, *Streptomyces griseus* EpoA, *Streptomyces coelicolor* SLAC, *Streptomyces sviceus* Ssl1, *B*. *subtilis* CotA, *Streptomyces lavendulae* REN-7 laccase, *T*. *thermophilus Tth* laccase, and *Thermobaculum terrenum* TtMCO.

### Purification and structural properties

**Fig 2:**
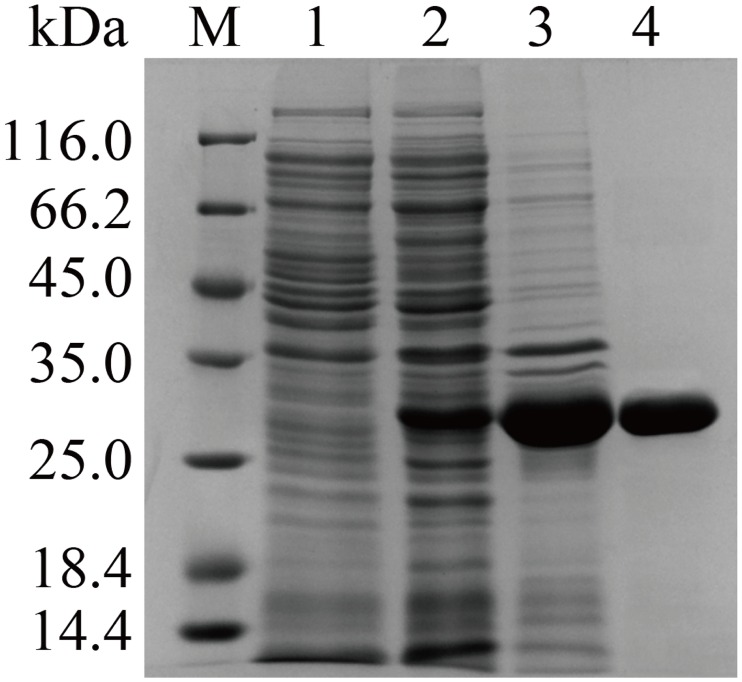
SDS-PAGE analysis of purified LaclK. M: molecular weight marker; lane 1: pET 28a vector plasmid (control); lane 2: supernatant of the sonication product; lane 3: precipitate of the sonication product; lane 4: LaclK purified via Ni–NTA.

After purification, a clear single protein band of approximately 30 kDa was detected by SDS-PAGE (Fig 2), which corresponds to the predicted value of the recombinant protein. UV-visible spectrum results (S1 Fig) indicated that LaclK lacks the traditional absorption maximums at 610 nm and 330 nm, characteristic of typical laccases, and the ratio of copper atoms/molecule of LaclK was calculated to be 0.86 ± 0.04. These spectral properties and metal contents of LaclK indicate that it is not a typical blue multicopper oxidase, but it also appears to differ from certain “yellow” or “white” laccases [5, 6, 22].

### Substrate specificity

Conventional laccase substrates such as 2,6-dimethoxyphenol (2,6-DMP), 2,2`-azino-bis(3-ethylbenzothiazoline-6-sulfonate) (ABTS), syringaldazine (SGZ), guaiacol and l-dopamine were used to evaluate the oxidation ability of LaclK. l-dopamine and 2,6-DMP could serve as substrates of LaclK, while SGZ, ABTS and guaiacol were not oxidized by LaclK. LaclK activity towards tyrosine was also tested, and no activity was detected.

The kinetic properties of LaclK for the substrates 2,6-DMP and l-dopamine were estimated at 85°C (pH 7.0) and 70°C (pH 6.0), respectively. The corresponding results are listed in Table 2. The low *k*_cat_ and *k*_cat_/*K*_m_ values were identified for 2,6-DMP and l-dopamine, indicating that LaclK lacks a strong ability to catalyze reactions with these substrates.

**Table 2:** Substrate specificity of LaclK.

Substrate	Optimum pH	*K*_m_ (mM)	*k*_cat_ (s^−1^)	*k*_cat_/*K*_m_ (mM^−1^ s^−1^)
2,6-DMP	7.0	0.457	0.468	1.024
l-dopamine	6.0	0.230	2.666	11.59

### Effects of pH and temperature on LaclK activity and stability

**Fig 3:**
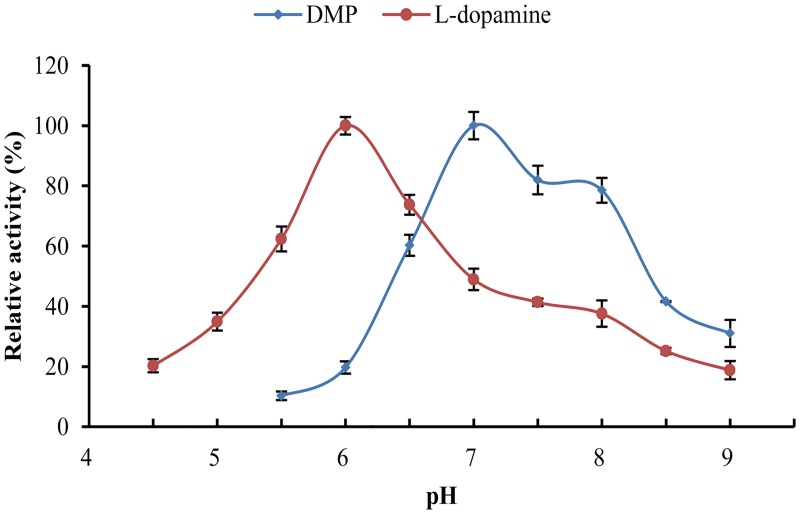
Effects of pH on the activity of LaclK.

The optimal pH for oxidation reactions varies for various substrates [4, 23]. As shown in Fig 3, the optimal pH values for LaclK activity towards the substrates l-dopamine and 2,6-DMP were 6.0 and 7.0, respectively. LaclK was quite stable under neutral to alkaline conditions (pH 7.0–8.0), more than 80% of its initial activity was retained after incubation for 6 days at pH 7.0 and 60°C; and there was no loss of activity after incubation for 7 days at pH 8.0 and 4°C (data not shown). Furthermore, LaclK activity was detectable after 60 days at pH 8.0 and 4°C.

**Fig 4:**
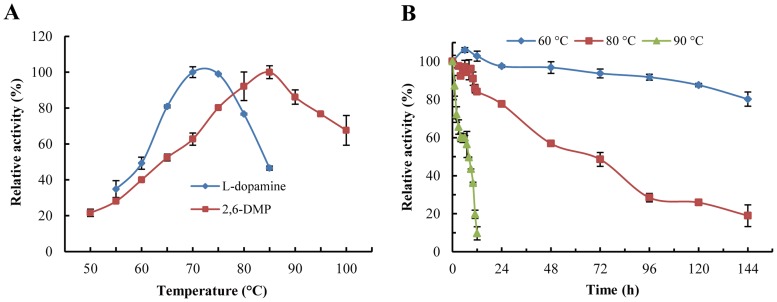
Effects of temperature on the activity and stability of LaclK. (A) Optimum temperature for enzyme activity. (B) Thermostability of LaclK at 60, 80 and 90°C with 2,6-DMP as the substrate at pH 7.0.

The most notable feature exhibited by LaclK is its high thermostability. Maximum LaclK activity were observed at 70°C and 85°C with l-dopamine and 2,6-DMP as substrates, respectively (Fig 4A). LaclK was highly stable at 60°C, retaining more than 80% of its initial activity after 6 days at pH 7.0 (Fig 4B). The half-life of LaclK at pH 7.0 and 80°C approximately72 h, and it was approximately 8 h at 90°C (Fig 4B). Interestingly, there was approximately 60% of its original activity was retained even at 100°C for 60 min.

### Effects of metal ions, inhibitors and organic solvents on LaclK activity

**Fig 5:**
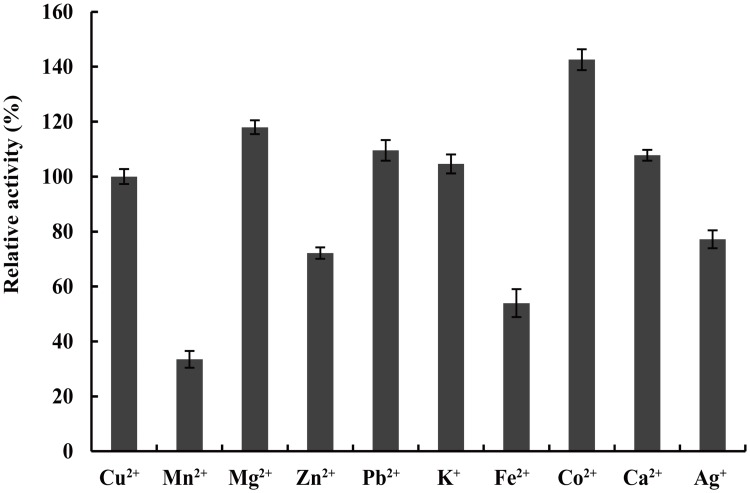
Effects of metal ions on LaclK activity. Enzyme activity was measured at 65°C in 50 mM Na_2_HPO_4_-KH_2_PO_4_ buffer (pH 7.0, supplemented with 0.2 mM CuSO_4_·5H_2_O) containing 1 mM metal ions and 2 mM 2,6-DMP.

The effects of 1 mM metal ions on LaclK activity were also assessed. As shown in Fig 5, activity was increased in the presence of Mg^2+^, Pb^2+^, K^+^, Co^2+^ or Ca^2+^, and Mn^2+^, Zn^2+^, Fe^2+^, or Ag^+^ showed inhibition effects on its activity. Performance stimulation trended as follows: Co^2+^ > Mg^2+^ > Pb^2+^ > Ca^2+^ > K^+^. Activity was stimulated up to 142% by 1 mM Co^2+^, and a similar obvious activity promoting by Co^2+^ was reported in SN4LAC laccase [13]. On the other hand, activity was reduced to 33%, 72%, 54%, and 77% in the presence of Mn^2+^, Zn^2+^, Fe^2+^, or Ag^+^, respectively. The inhibitory effects of Mn^2+^, Zn^2+^, Fe^2+^, or Ag^+^ were generally observed in other laccases [13, 24]. Copper is a component of active site of laccases, Cu^2+^ was essential for substrate oxidation, and 0.2 mM Cu^2+^ was found to be optimal for LaclK activity (S2 Fig).

The sensitivity of LaclK to several putative laccase inhibitors was also evaluated. Purified LaclK was strongly inhibited by l-cysteine (l-Cys), ethylene diamine tetraacetic acid (EDTA) and dithiothreitol (DTT) (Table 3); these compounds also demonstrated strong inhibition for other fungal or bacterial laccases [4, 24, 25]. Sodium dodecyl sulfate (SDS) at a low concentration (1 mM) slightly stimulated LaclK activity, similar to reports of other phenol oxidases [1, 26]. In contrast, LaclK exhibited high tolerance to the inhibitor sodium azide (NaN_3_), retaining 81% of its original activity at 10 mM NaN_3_. For chloride salt, LaclK activity decreased as the NaCl concentration increased. The enzyme was 90% active in the presence of 100 mM NaCl, while the enzyme retained approximately 19% of its activity at a 1000 mM concentration of NaCl, indicating that LaclK is sensitive to NaCl.

**Table 3:** Effects of inhibitors on LaclK activity.

Inhibitors	Concentration (mM)	Relative activity (%)
Control	-	100 ± 2.82
SDS	1	105.25 ± 4.57
	5	49.11 ± 3.16
	10	15.49 ± 1.72
L-Cys	0.1	92.45 ± 3.20
	0.5	64.11 ± 2.56
	1	10.80 ± 1.33
EDTA	0.1	82.42 ± 4.28
	0.5	5.20 ± 1.09
	1	0
NaN_3_	0.5	93.28 ± 4.08
	5	91.62 ± 2.10
	10	81.99 ± 3.72
DTT	0.1	77.21 ± 1.46
	0.5	3.55 ± 0.65
	1	0
NaCl	100	90.50 ± 6.72
	200	62.31 ± 2.38
	500	28.27 ± 2.35
	1000	19.20 ± 1.26

When we tested the influence of several solvents on LaclK activity, we found that all solvents tested exerted positive effects on enzyme activity in the presence of 10% organic solvent (v/v) (Table 4). Remarkably, LaclK retained approximately the same original activity, even in the presence of 30% butyl alcohol.

**Table 4:** Effects of various solvents on LaclK activity.

Solvent	Concentration (%, v/v)	Relative activity (%)
Control	-	100 ± 3.07
Methanol	10	102.14 ± 5.68
	20	94.80 ± 3.78
	30	91.57 ± 2.35
Ethanol	10	125.39 ± 5.18
	20	90.79 ± 2.18
	30	57.83 ± 1.79
Isopropyl alcohol	10	137.16 ± 6.38
	20	94.21 ± 4.33
	30	41.59 ± 2.37
Butyl alcohol	10	112.91 ± 3.55
	20	132.30 ± 4.29
	30	124.17 ± 5.02
Triton x-100	10	156.85 ± 6.52
	20	100.38 ± 4.43
	30	58.27 ± 2.76
Dimethyl sulfoxide	10	153.23 ± 5.48
	20	31.35 ± 1.55
	30	22.95 ± 1.86

### Dye decolorization

**Fig 6:**
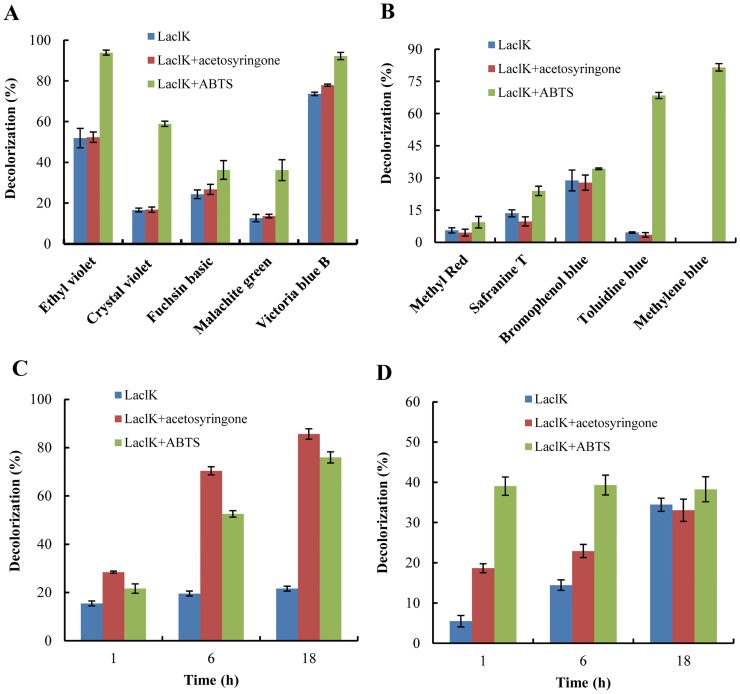
Decolorization of synthetic dyes by LaclK. (A) Decolorization of five triphenylmethane dyes (25 μM) by LaclK with or without mediators at 60°C for 1 h. (B) Decolorization of an azo dye (methyl red, 25 mg/L) and four phenolic dyes (25 mg/L) by LaclK with or without mediators at 60°C for 1 h. (C) Decolorization of Congo Red (25 mg/L) after different incubation times with or without mediators at 60°C. (D) Decolorization of Brilliant Green (25 mg/L) after different incubation times with or without mediators at 60°C.

The ability of LaclK to decolorize chemically diverse dyes was investigated in this study. LaclK was able to decolorize all tested dyes in the presence of a mediator. The addition of 0.1 mM ABTS notably increased the decolorization efficiency for all dyes (Fig 6), and more than 80 or 90% decolorization was observed for Ethyl Violet, Victoria Blue B and Methylene Blue after incubation for 1 h (Fig 6A and 6B). Moreover, ABTS was necessary for the efficient decolorization of Methylene Blue (Fig 6B). The promotion effects of mediators in dye decolorization have also been demonstrated by other researchers [26–28].

However, the addition of acetosyringone did not appear to increase the decolorization of triphenylmethane dyes (Fig 6A); yet, acetosyringone exerted slight inhibitory effects on the decolorization of toluidine blue, methyl red, safranine T and bromophenol blue (Fig 6B). Conversely, acetosyringone exhibited more notable effects than ABTS on Congo Red decolorization (Fig 6C). It appears that the optimal mediator for dye decolorization is dye-dependent, and ABTS may be the best mediator for LaclK for the decolorization of triphenylmethane dyes.

The decolorization increased with increasing incubation time. Only 19% of brilliant green and 28% of Congo Red was decolorized after 1 h in the presence of acetosyringone; and the highest decolorization, observed after 18 h, was approximately 33% for brilliant green (Fig 6D) and 86% for Congo Red (Fig 6C).

## Discussion

The laccases have been widely employed in industrial and environmental applications due to their wide spectrum of catalyzed reactions [9]. With rising demand for novel laccases that exhibit extensive pH-range tolerance and high-temperature stability, more enzymes of bacterial origin have been investigated [4, 11–13]. Based on bioinformatic analyses and characterization of the dye decolorization ability of *K*. *huakuii* LAM0618^T^, and considering that these features likely correlate with laccase function, the putative laccase-like gene *laclK* was cloned, overexpressed in *E*. *coli* and characterized for the first time.

We found that LaclK could oxidize typical laccase substrates, but it exhibited structural and catalytic properties that were distinct from other laccases. Four histidine-rich copper binding domains are a common characteristic of laccases, and four copper atoms per active protein monomer are essential for laccase activity [2]. However, the identification of white laccases (containing one copper atom, one iron/manganese atom and two zinc atoms) [22] and the RL5 laccase (lacking four histidine-rich copper binding domains but containing four copper atoms) [8] has suggested that a novel method define laccases is necessary. The copper content of LaclK was determined to consist of one, rather than four, copper atoms per LaclK molecule according to atomic absorbance spectrometry. In terms of molecular mass, LaclK is similar to Tfu1114 and RL5 but lower than regular three-domain laccases and two-domain laccase. However, the catalytic properties of LaclK differ from RL5, Tfu1114, and other classical bacterial laccases [8, 13, 19, 26]. The *k*_cat_ values of LaclK are similar to certain laccase-like multicopper oxidases [29–31] but are significantly lower than those previously reported for laccases [8, 26]. The distinct structural characteristics of LaclK may account for its catalytic properties; thus, further experimental characterization of LaclK is necessary.

EDTA acts as a specific divalent cation chelator [32]. LaclK activity was completely inhibited by 1 mM EDTA, suggesting that Cu^2+^ is essential for LaclK activity and that LaclK should be a copper-containing enzyme. LaclK exhibited no maximum at 330 nm, implying that LaclK may lack type 3 coppers [6]. For an effective laccase inhibitor, activity inhibition occurs when NaN_3_ binds type 2 and 3 copper centers in laccases [33]. Tolerance to NaN_3_ indicated that the copper centers of LaclK may not be available to NaN_3_. Coincidentally, Tfu1114 exhibited similar insensitivity to NaN_3_, and the copper content of Tfu1114 was also determined to consist of one rather than four copper atoms [19]. Although copper depletion from laccases upon overexpression in *E*. *coli* was observed previously [11, 34, 35] and weak incorporation between LaclK and copper atoms may lead to copper removal during the ultrafiltration step, and even given that LaclK is assigned to the multi-copper polyphenol oxidoreductase laccase family, it is rather difficult to classify LaclK as a new laccase because the copper center is distinct from reported laccases and much lower catalytic efficiency towards laccase substrates was observed. Given its structural and catalytic properties, LaclK represents a novel, uncharacterized copper-containing enzyme that exhibits laccase activity.

The optimal pH for LaclK is substrate-dependent, and bell-shaped activity profiles for substrates (Fig 3) were observed. It is well known that most fungal laccases are functional under acidic to neutral pH conditions but lose their activity under alkaline conditions [36], whereas bacterial laccases usually function under neutral to alkaline pH conditions [4]. LaclK oxidized l-dopamine and 2,6-DMP under neutral to alkaline pH conditions, which is generally observed for bacterial laccases [13, 34, 37]. The solvent stability of enzymes is thought to positively correlate with thermostability [38]. In this work, we demonstrated a correlation between the thermostability of LaclK and its tolerance of organic solvents. The half-life of LaclK (72 h) at 80°C was 5-fold higher than that of the *Tth* laccase, demonstrating the extreme robustness of LaclK. In addition, tolerance to organic solvents confers many attractive advantages upon LaclK, which will be beneficial for the potential applications of LaclK under harsh industrial process conditions [38, 39]. Generally, the host organisms of thermostable enzymes exhibit optimal growth temperatures higher than 45°C [10]. The growth temperature range for *K*. *huakuii* LAM0618^T^ is 10°C to 45°C (with optimal growth at 30°C) [16]. How does a moderately thermophilic bacterium possess such a highly thermostable enzyme? What is the mechanism underlying the stability of LaclK? Furthermore, what is the physiological role of LaclK in *K*. *huakuii* LAM0618^T^? Such issues are of particular interest.

To better understand the thermostability of LaclK, the aliphatic indices and instability indices of LaclK and certain previously reported heat-resistant laccase or laccase-like oxidases were analyzed (Table 1). The aliphatic index of recombinant LaclK (75.62) is lower than the indices of CotA (77.89), REN-7 (79.18), TtMCO (81.61) and the *Tth* laccase (96.39) [12, 30, 35, 40]. However, recombinant LaclK (31.92) demonstrated the lowest instability index among CotA (45.51), REN-7 (42.86), TtMCO (47.45) and the *Tth* laccase (42.21). Conversely, although the aliphatic indices of EpoA (66.19), SLAC (61.81) and Ssl1 (67.82) were calculated to be very low, high stability was observed for these enzymes [11, 41, 42]. Low instability indices (34.70 for EpoA, 29.63 for SLAC, 31.78 for Ssl1) may contribute to enzyme stability under harsh conditions. We also found that enzymes with low molecular weight (per monomer) possess low instability indices, and these enzymes are more likely to be resistant or stable enzymes. The parameters calculated for LaclK are similar to Tfu1114. Tfu1114 was reported to be stable against organic solvents and heat, with a half-life of 4.7 h at 90°C [19]. These results support our findings that LaclK is highly thermostable and organic solvent-tolerant. Admittedly, the factors affecting protein thermostability or stability can be complicated, and there is no single physicochemical factor that can account for the stability profile of a protein [10, 21]; therefore, our predicted results must be experimentally verified.

It has been reported that a number of fungal and bacterial laccases are able to decolorize and degrade industrial dyes [4, 25–27, 33, 43]. Triphenylmethane dyes rank as the third most widely used textile dyes after azo dyes and anthraquinone dyes. Over the past few decades, the application of laccases to decolorize dyes has been extensively studied [4, 25–27, 33, 43]. However, the potential of proteins from the Cu-oxidase_4 superfamily to degrade dyes remains unknown. Our investigation is the first to demonstrate the ability of LaclK to decolorize triphenylmethane dyes and aromatic dyes as well as azo dyes. Thus, its wide-spectrum decolorization ability suggests that LaclK is an alternative for the biological treatment of industrial dye-containing effluents.

## Conclusions

A novel copper-containing polyphenol oxidase from the Cu-oxidase_4 superfamily has been purified and characterized. LaclK exhibited laccase activity and dye decolorization ability, while its structural and catalytic properties were distinct from laccases and laccase-like proteins that have been reported previously. Furthermore, LaclK is extremely thermostable and organic solvent-tolerant. These unusual properties of LaclK suggest that it is an alternative for industrial applications, contribute to our understanding of the diversity of laccase-like proteins and raise scientific interest in LaclK protein engineering studies to further improve the application of laccases in various fields.

## Materials and Methods

### Strains and Chemicals

Strain *K*. *huakuii* LAM0618^T^ was provided by the Agricultural Culture Collection of China (ACCC 06121). *E*. *coli* strains DH5α and BL21 (DE3) (Tian Gen Co., Ltd, China) were used as host strains for plasmid propagation and protein expression, respectively. The *E*. *coli* strains were routinely grown in Luria-Bertani medium. Antibiotics were added at desired concentrations (50 μg/mL kanamycin or 100 μg/mL ampicillin). A genomic DNA kit, a gel extraction kit and a plasmid kit were purchased from Omega Bio-Tek (Norcross, GA, USA). Enzymes for cloning procedures and protein ladders were obtained from Fermentas (St. Leon-Rot, Germany). ABTS, SGZ, 2,6-DMP, guaiacol and l-dopamine were purchased from Sigma-Aldrich (St. Louis, MO, USA). Other chemicals were of analytical grade or higher and were purchased from commercial sources.

### Sequence analysis of *laclK*

The open reading frame (ORF) of *laclK* was predicted using NDAMAN 8. Sequence searching and putative conserved domain detection were carried out using the BlastP program at NCBI. The aliphatic index and the instability index were computed using the ProtParam tool (http://web.expasy.org/protparam/). Multiple sequence alignment was performed with the Clustal W program.

### Cloning of the *laclK* gene

The *laclK* gene (NCBI accession no. WP_029500662) was amplified by PCR with the primers *laclK*-F (5ʹ-CGC GGATCC ATGACAACAACAATTTATACG-3ʹ) and *laclK*-R (5ʹ-CCG CTCGAG TTACTTTCGCACGATAAAGCT-3ʹ) using genomic DNA from *K*. *huakuii* LAM0618^T^ as the template. *BamHI* and *XhoI* endonuclease recognition sites are underlined. The PCR product was purified and cloned into the pZero Back/Blunt Vector, and then the recombinant plasmid and pET28a vector were digested with the same endonucleases *(BamH*I and *Xho*I). The digestion products were ligated with T4 ligase, resulting in the expression plasmid pET28a-*laclK*. The nucleotide sequence of the insert was confirmed by sequencing (Life Technologies Company, Beijing, China).

### Expression and purification of recombinant LaclK

*E*. *coli* BL21 (DE3) cells harboring pET28a-*laclK* were grown at 37°C and 180 r/min until an optical density of 0.6 at 600 nm (OD_600_) was reached; then, 0.2 mM isopropylβ-D-thiogalactoside (IPTG) and 0.2 mM CuSO_4_·5H_2_O were added to the culture medium, and the temperature and rotation speed were reduced to 16°C and 120 r/min, respectively. After continued cultivation for 16 h, the culture was harvested by centrifugation (5000 ×g at 4°C for 10 min) and lysed by sonication in cold lysis buffer (50 mM Tris-HCl, pH 8.0, 300 mM NaCl, 10 mM imidazole). To remove cell debris, lysate was centrifuged at 14,000 ×g at 4°C for 30 min. The supernatant (crude extract) was loaded onto a Ni-NTA column (QIAGEN, Germany), resulting in binding of the target protein to Ni-NTA. The NTA column was washed at least twice with wash buffer (50 mM Tris-HCl, pH 8.0, 300 mM NaCl, 20 mM imidazole). Then, recombinant LaclK was eluted with elution buffer (50 mM Tris-HCl, pH 8.0, 300 mM NaCl, 300 mM imidazole) [26]. Subsequently, NaCl and imidazole were removed from the eluted fraction by ultrafiltration with 50 mM Tris-HCl buffer (pH 8.0). The purity of LaclK was analyzed by sodium dodecyl sulfate polyacrylamide gel electrophoresis (SDS-PAGE). Protein concentration was determined using the BCA method. The purified LaclK samples were stored at 4°C or preserved in 25% (v/v) glycerol at −20°C until further use.

### Enzyme assay

LaclK laccase activity was assayed using l-dopamine and 2,6-DMP (dissolved in anhydrous ethanol) as substrates. The oxidation of 2 mM l-dopamine was detected at 70°C and 475 nm (ɛ_475_ = 2835 M^-1^ cm^-1^) in 50 mM Na_2_HPO_4_-KH_2_PO_4_ buffer (pH 6.0). The oxidation of 2,6-DMP (2 mM) was determined at 85°C and 468 nm (ɛ_468_ = 49,600 M^-1^ cm^-1^) in 50 mM Na_2_HPO_4_-KH_2_PO_4_ buffer (pH 7.0). Alternative substrates, 0.1 mM SGZ (ɛ_525_ = 65,000 M^-1^ cm^-1^), 0.5 mM ABTS (ɛ_420_ = 36,000 M^-1^ cm^-1^) and 2 mM guaiacol (ɛ_465_ = 12,000 M^-1^ cm^-1^) were employed to determine LaclK activity [43]. LaclK activity towards 0.1 mM tyrosine (ɛ_280_ = 4400 M^-1^ cm^-1^) was tested at pH 4.0, 7.0 and 9.0 [11]. The reaction mixture contained appropriately diluted enzyme, pH buffer, substrates and 0.2 mM Cu^2+^. After incubation at 70°C for 1min (for l-dopamine) or 85°C for 3 min (for 2,6-DMP), the mixture was transferred to an ice-water bath for 1 min to stop the reaction, and the absorbance was measured. Enzyme activity measurements were performed with a DU800 Nucleic Acid/Protein Analyzer (Beckman Coulter, USA). One unit (U) of enzyme activity was defined as the amount of enzyme needed to catalyze 1 μmol of substrate per minute. All assays were performed at least three times.

### Characterization of LaclK

The effects of pH on LaclK activity were determined in sodium citrate buffer (pH 4.5 to 5.5), Na_2_HPO_4_-KH_2_PO_4_ buffer (pH 5.5 to 8.0), and Tris-HCl buffer (pH 8.0 to 9.0). The optimal temperature for activity was assessed at temperatures ranging from 50 to 100°C by measuring l-dopamine and 2,6-DMP oxidation. Thermostability of the enzyme was measured in 50 mM Na_2_HPO_4_-KH_2_PO_4_ buffer (pH 7.0) at 60, 80 and 90°C using 2,6-DMP as a substrate.

The effects of metal ions on LaclK activity were determined by incubating Ca^2+^, Co^2+^, Cu^2+^, Fe^2+^, Mg^2+^, Mn^2+^, Pb^2+^, Ag^+^, Zn^2+^, and K^+^ with LaclK at 4°C for 30 min prior to the addition of 2,6-DMP. The effects of different concentrations of inhibitors and organic solvents on enzyme activity were studied using 2,6-DMP as a substrate by adding each factor with LaclK at 4°C for 30 min prior to the determination of residual activity. Activities assayed were determined at 65°C and pH 7.0 in the presence of 0.2 mM Cu^2+^ but in the absence of the reagent was considered 100%.

Kinetic parameters for LaclK were determined using different concentrations of 2,6-DMP (0–10 mM) and l-dopamine (0–10 mM). The total copper content of LaclK was determined by atomic absorption spectroscopy as previously described [44]. The UV-visible absorption spectrum of purified LaclK (13 μM) was measured in the range of 300–800 nm in 50 mM Tris-HCl buffer (pH 8.0) on a UV-1800 UV-VIS spectrophotometer (Shimadzu, Tokyo, Japan).

### Dye decolorization

Six triphenylmethane dyes (25 μM), two azo dyes (25 mg/L) and four aromatic dyes (25 mg/L) were selected to evaluate the ability of LaclK to decolorize structurally different dyes (S1 Table). Decolorization was tested in the presence or absence of mediator (ABTS or acetosyringone). The reaction mixture (1 mL) contained the dye, LaclK (60 U/L), a mediator (0.1 mM), Cu^2+^ (0.2 mM) and the appropriate buffer. The specific activity of purified LaclK was 0.63 U mg^−1^ for the oxidation of 2,6-DMP at 65°C and pH 7.0. Reactions were initiated by adding LaclK, and mixtures were incubated at 60°C without shaking in the dark. The control samples were assessed in parallel without the addition of LaclK. Dye decolorization progress was measured 1, 6 and 18 h after incubation started. The decolorization percentage was calculated based on the relative decrease in the absorbance at the maximal absorbance wavelength for each dye.

## Supporting Information

S1 Fig UV/Vis spectrum of LaclK in 50 mM Tris-HCl buffer (pH 8.0). (TIF)

S2 Fig Copper dependence of activity. Enzymatic activity was measured at 65°C in 50 mM Na_2_HPO_4_-KH_2_PO_4_ buffer (pH 7.0) containing 2 mM 2,6-DMP. The results indicated that Cu^2+^ is essential for 2,6-DMP oxidation, and a concentration of approximately 0.2 mM Cu^2+^ was found to be optimal for the activity of purified LaclK.
(TIF)

S1 Table Information regarding dye types and tested conditions. (DOCX)
