# Supplementary figures and images for: Characterization of a Highly Thermostable and Organic Solvent-Tolerant Copper-Containing Polyphenol Oxidase with Dye-Decolorizing Ability from Kurthia huakuii LAM0618T
Source: PLoS One. 2016 Oct 14;11(10):e0164810. doi: 10.1371/journal.pone.0164810 (PMC5065135; doi:10.1371/journal.pone.0164810)

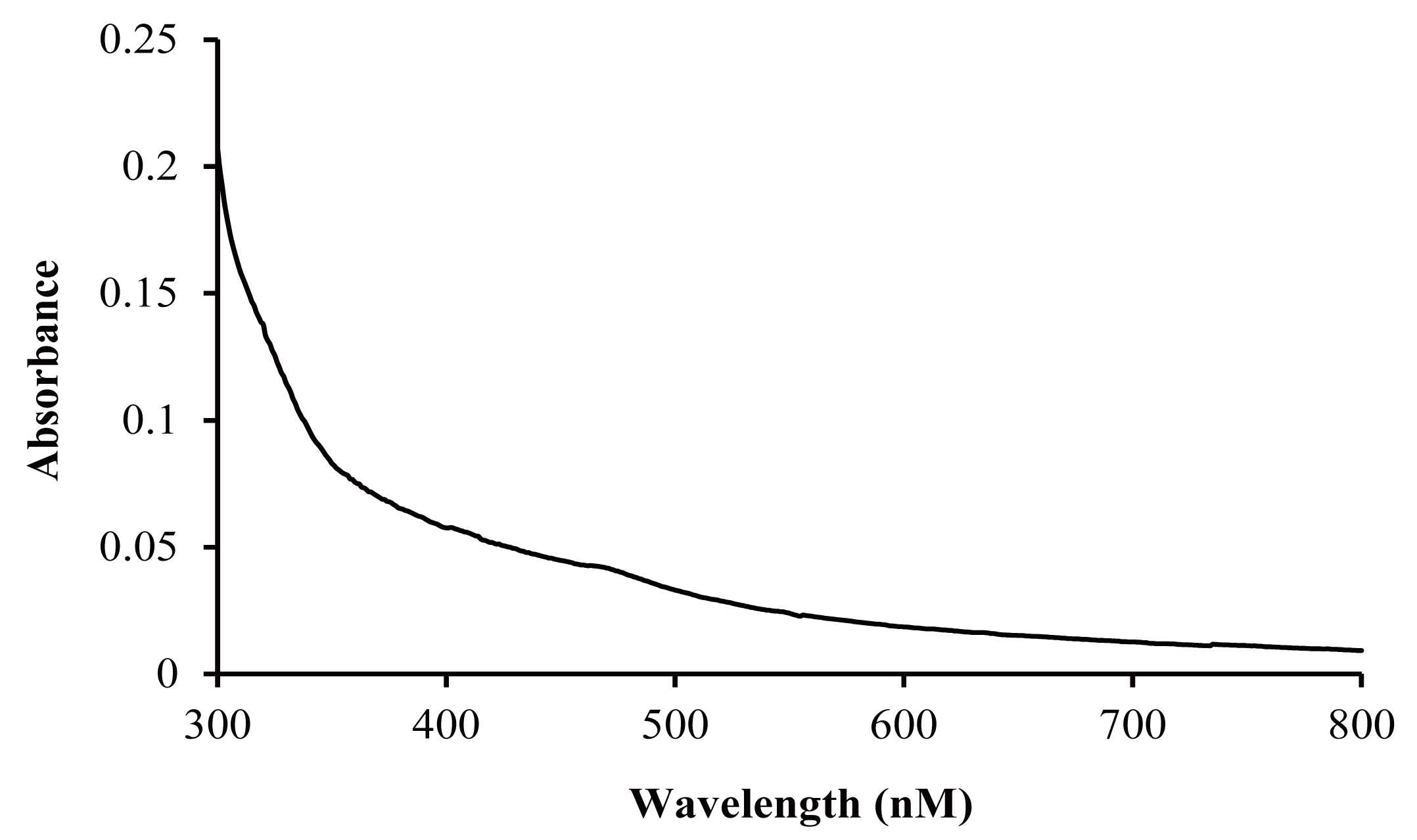

Supplement: S1 Fig — (TIF) [file pone.0164810.s001.tif]

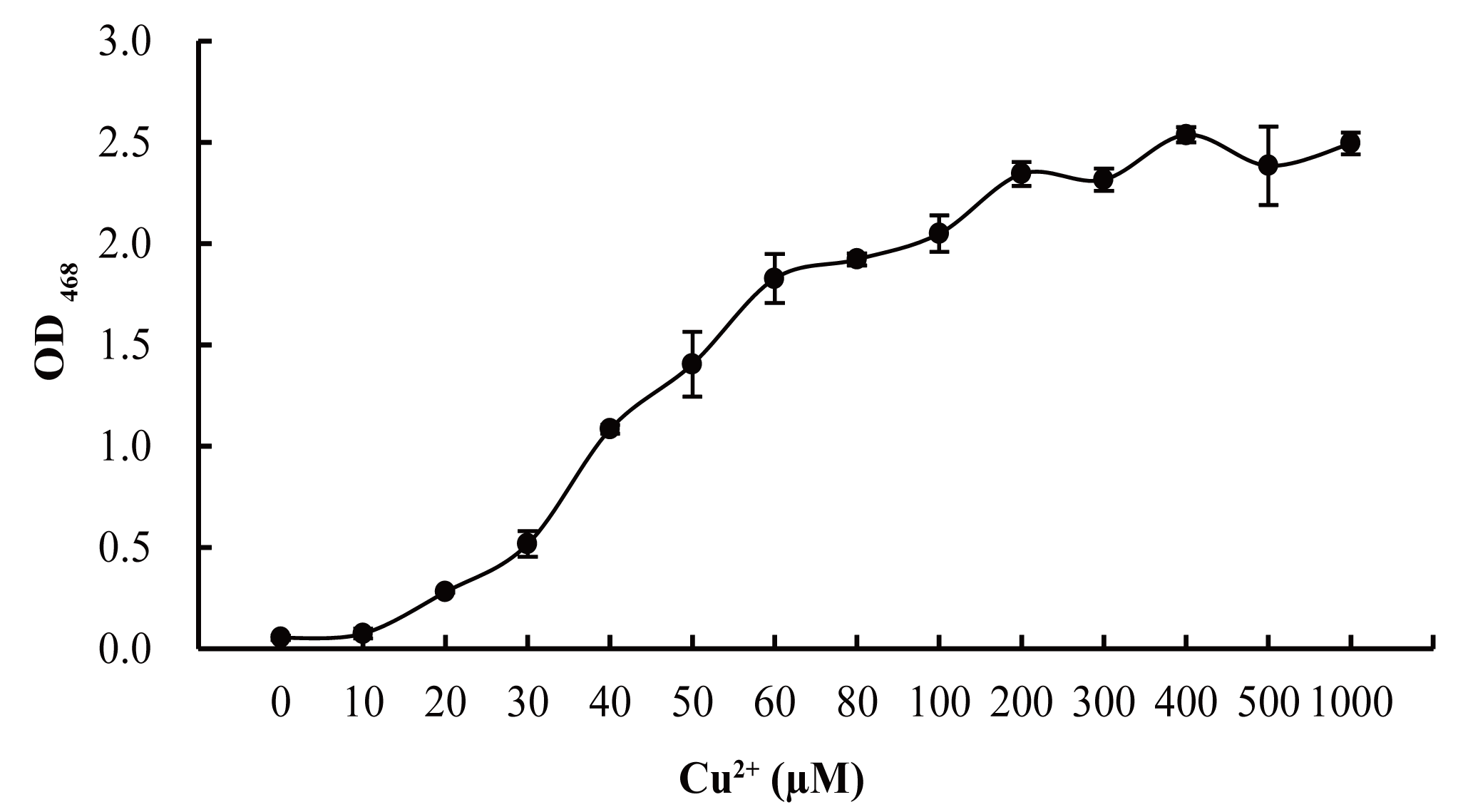

Supplement: S2 Fig — Enzymatic activity was measured at 65°C in 50 mM Na2HPO4-KH2PO4 buffer (pH 7.0) containing 2 mM 2,6-DMP. The results indicated that Cu2+ is essential for 2,6-DMP oxidation, and a concentration of approximately 0.2 mM Cu2+ was found to be optimal for the activity of purified LaclK. (TIF) [file pone.0164810.s002.tif]
