# Supplementary material for: Characterization of a Highly Thermostable and Organic Solvent-Tolerant Copper-Containing Polyphenol Oxidase with Dye-Decolorizing Ability from Kurthia huakuii LAM0618T
Source: PLoS One. 2016 Oct 14;11(10):e0164810. doi: 10.1371/journal.pone.0164810 (PMC5065135; doi:10.1371/journal.pone.0164810)
Supplement: S1 Table — (DOCX) [file pone.0164810.s003.docx]

**S1 Table. Information regarding dye types and tested conditions.**

| **Classification** | **Dyes** | **Test pH** | **Absorbance wavelength (nm)** |
| --- | --- | --- | --- |
| Triphenyl methane dyes | Malachite green | 5.0 | 617 |
|  | Crystal violet | 7.0 | 590 |
|  | Fuchsin basic | 5.0 | 543 |
|  | Ethyl violet | 7.0 | 596 |
|  | Brilliant green | 6.0 | 623 |
|  | Victoria blue B | 6.0 | 599 |
| Azo dyes | Congo red | 7.0 | 488 |
|  | Methyl red | 7.0 | 410 |
| Other aromatic dyes | Bromophenol blue | 7.0 | 422 |
|  | Safranine T | 7.0 | 530 |
|  | Methylene blue | 7.0 | 664 |
|  | Toluidine blue | 7.0 | 620 |
